# Changes in fruit and vegetable consumption habits from pre-pregnancy to early pregnancy among Norwegian women

**DOI:** 10.1186/s12884-017-1291-y

**Published:** 2017-04-04

**Authors:** Marianne Skreden, Elling Bere, Linda R. Sagedal, Ingvild Vistad, Nina C. Øverby

**Affiliations:** 1 Department of Public Health, Sports and Nutrition, University of Agder, PO Box 422, 4604 Kristiansand, Norway; 2 Department of Obstetrics and Gynaecology, Sørlandet Hospital HF, PO Box 416, 4604 Kristiansand, Norway; 3 Department of Research, Sørlandet Hospital HF, PO Box 416, 4604 Kristiansand, Norway

**Keywords:** Fruits, Preconception, Pregnancy, Vegetables

## Abstract

**Background:**

A healthy diet is important for pregnancy outcome and the current and future health of woman and child. The aims of the study were to explore the changes from pre-pregnancy to early pregnancy in consumption of fruits and vegetables (FV), and to describe associations with maternal educational level, body mass index (BMI) and age.

**Methods:**

Healthy nulliparous women were included in the Norwegian Fit for Delivery (NFFD) trial from September 2009 to February 2013, recruited from eight antenatal clinics in southern Norway. At inclusion, in median gestational week 15 (range 9–20), 575 participants answered a food frequency questionnaire (FFQ) where they reported consumption of FV, both current intake and recollection of pre-pregnancy intake. Data were analysed using a linear mixed model.

**Results:**

The percentage of women consuming FV daily or more frequently in the following categories increased from pre-pregnancy to early pregnancy: vegetables on sandwiches (13 vs. 17%, *p* <0.01), other vegetables (11 vs. 14%, *p* = 0.01), fruits (apples, pears, oranges or bananas) (24 vs. 41%, *p* < 0.01), other fruits and berries (8 vs. 15%, *p* < 0.01) and fruits and vegetables as snacks (14 vs. 28%, *p* < 0.01). The percentage of women who reported at least daily consumption of vegetables with dinner (22% at both time points) was stable. A higher proportion of older women increased their consumption of vegetables and fruits as snacks from pre-pregnancy to early pregnancy compared to younger women (*p*=0.04).

**Conclusions:**

We found an increase in the proportion of women consuming FV daily or more frequently from pre-pregnancy to early pregnancy.

**Trial registration:**

ClinicalTrials.gov database, NCT01001689. https://clinicaltrials.gov/ct2/show/NCT01001689?term=NCT01001689&rank=1.

## Background

Maternal diet pre-conception and during pregnancy may influence pregnancy outcome [1–5], and the future health of mother and child [6–8]. Plant-based dietary patterns which contain a variety of fruit and vegetables (FV) are associated with reduced risk of congenital anomalies [9], preterm birth [3], and more favourable foetal growth [1–3, 5, 10], as well as lower frequency of maternal complications such as excessive gestational weight gain [3], preeclampsia [4] and gestational diabetes [11].

Because of their high content of micronutrients, fibre and other bioactive compounds such as phytochemicals [12], FV are essential parts of a healthy and balanced diet. Thus, increasing the consumption of FV is an important public health goal [13, 14]. The Norwegian National Health Authorities advocate a dietary pattern rich in FV to the general population as well as to pregnant women. They recommend a minimum daily intake of five servings or 500 g of FV, of which at least one half should be vegetables [15]. Although there has been an increase in the intake of FV over the last decades in Norway [16], large differences in intake of FV are described across different population groups [17]. Despite public health recommendations [15], only 15% of Norwegian women were found to achieve the recommended amount of 250 g vegetables per day, whereas 41% reported to have an intake of at least 250 gram of fruit (including 100 ml of fruit juice) in a national survey conducted in 2010–2011 [17]. Furthermore, in the Norwegian Mother and Child Cohort Study (MoBa), only 33 and 7% of participating pregnant women reported reaching the recommended intake of fruits and vegetables, respectively [18]. The gap between recommendations and actual consumption is a global concern, both in low- and middle -income countries [19], and in more affluent parts of the world [18–20].

Several longitudinal studies have reported that overall dietary patterns remain relatively stable from pre-pregnancy and throughout pregnancy [21, 22]. However, women are known to increase their intake of fruits [21, 23, 24] from pre-pregnancy to the first half of pregnancy, whereas reports in changes in vegetables consumption are mixed [21, 23, 24]. Both the nutritional state in which women enter pregnancy and nutrition in pregnancy may influence pregnancy outcome and the health of the next generation. It is therefore important to identify particular groups of women with poor dietary habits, in order to provide more targeted interventions. The main aim of the present study was to explore the changes from pre-pregnancy to early pregnancy in consumption of FV, and to describe associations with maternal educational level, BMI, and age.

## Methods

### Population and study design

The present paper is based on data from the Norwegian Fit for Delivery (NFFD) study [25]. The NFFD is a randomised, blinded controlled trial performed in southern Norway. The intervention was a combination of antenatal counselling on diet and physical activity. The main aims of the NFFD trial are to examine the effect of the intervention on maternal gestational weight gain and postnatal weight retention, glucose regulation during pregnancy, newborn birth weight and the incidence of operative deliveries. The NFFD trial has previously been described in detail [25]. Between September 2009 and January 2013 midwives at eight local antenatal clinics consecutively recruited pregnant nulliparous women. Other eligibility criteria were age ≥ 18 years, singleton pregnancy at ≤ 20 + 6 weeks of gestation, pre-pregnancy BMI ≥ 19.0 kg/m^2^, that the woman was fluent in Norwegian or English and was able to provide informed consent. Exclusion criteria were on-going substance abuse, pre-existing diabetes mellitus, disabilities which precluded participation in a physical fitness program, and planned relocation outside the study area before delivery.

**Fig. 1:**
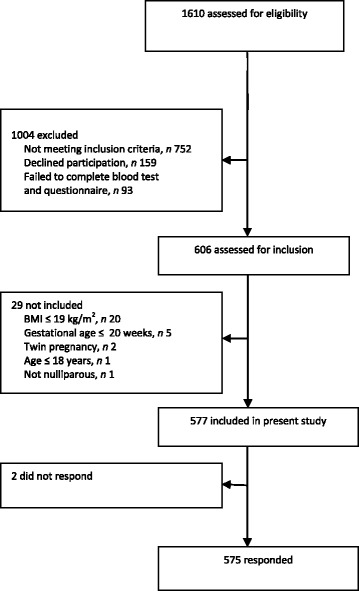
Flow diagram of the inclusion of pregnant women in the present study

Of 1610 nulliparous women, 606 were included into the study [26], and 575 participated in the present sub study (Fig. 1). The study design was cross-sectional. At inclusion, in median gestational-week 15.0 (range; 9.0–20.0), the women answered a 43-item food frequency questionnaire (FFQ) and reported how often they currently consumed the different foods, and in retrospect how often they ate the different foods before they got pregnant. Randomization took place after the women had answered the FFQ.

### Assessment of dietary changes

The questionnaire items on fruit- and vegetable-intake have previously been used for assessing intake of FV among schoolchildren [27] and included the following six items; “vegetables at dinner”, “vegetables on your sandwich”, “other vegetables” (for example, carrots at lunchtime), “fruits” (apples, oranges, pears or bananas), “other fruits or berries” (fruits or berries other than apples, oranges, pears or bananas), “fruits or vegetables as snacks”. The FFQ questions were; “How often do you eat…..now? and in retrospect “How often did you eat… before pregnancy?”. There were ten response alternatives which were recoded into frequency of consumption (“0 = never”, “0.5 = less than once a week”, “1 = weekly”, “2 = twice weekly”,….”6 = six times weekly”, “7 = daily” and “10 = several times daily”). FV consumption frequency was categorized into three groups: i) ≤ 1/week, ii) 2–6/week and ≥1/day. Furthermore, FV consumption frequency was categorized into two groups: “≥1/day” vs. “<1/day” to examine potential associations between change in FV consumption and the women’s educational level, BMI and age.

A test-retest reliability study was performed in a sample of 105 pregnant women who completed the presented questionnaire 2 weeks apart [28]. The six included items in this paper (both before and in pregnancy yielding 12 correlations) were found to have acceptable test-retest correlations (Pearson’s correlation coefficients) ranging from r = 0.525 (*p* = <0.01) for the variable “other fruits before pregnancy” to r = 0.800 (*p* = <0.01) for “fruits before pregnancy”.

### Other study variables

The questionnaire completed at inclusion also contained questions about lifestyle and background factors such as maternal educational level, pre-pregnancy BMI and age at inclusion. The following response options on level of education were; < 7 years of primary education; 7–10 years of primary education; trade school or 1–2 years of high school; completed high school; < 4 years at college/university and ≥ 4 years at college/university. Educational level was dichotomized into low education (not having attended college or university) and high education (having attended college or university). Height was measured, using a stadiometer (Seca Leicester, Hamburg, Germany). Pre-pregnancy weight was self-reported and used for calculation of pre-pregnancy BMI (weight/height^2^). In line with the World Health Organization’s definition of normal weight and overweight/obese [29], we dichotomized: BMI < 25 kg/m^2^ vs. BMI ≥ 25 kg/m^2^. Maternal age was dichotomized into < 25 years vs. ≥ 25 years.

### Statistical methods

All statistical analyses were performed with SPSS for IBM statistical software packages version 22.0 (IBM Corporation, NY, USA). A two-sided *p* value of 0.05 was considered significant. Differences between responders and non-responders were tested with Pearson Chi-square test for categorical data and Student’s *t* test for continuous variables. FV consumption and the changes in FV consumption from pre-pregnancy to median gestational week 15.0 were analysed using a linear mixed model with dichotomized FV consumption as the dependent variable [30]. Differences in pregnant women’s FV consumption might be dependent on educational level, BMI and age [8, 31–36]. Accordingly, the model included maternal educational level, BMI and age, as well as the following interaction terms: time^*^maternal education, time^*^BMI and time^*^age, to investigate potential difference in changes in the consumption of FV from pre-pregnancy to median gestational week 15 between low and high educational level, BMI and age. A *p* value of the product term of less than 0.10 was defined as a significant effect.

## Results

### Sociodemographic characteristics

The inclusion of pregnant women is shown in Fig. 1. Sociodemographic characteristics of the 575 women who were included and answered the FFQ are reported in Table 1. Mean maternal age was 28.1 (SD 4.4) years, and mean pre-pregnancy BMI was 23.9 kg/m^2^ (SD 3.8). Pre-pregnancy, a larger proportion of women with higher educational attainment reported higher frequency in consumption of vegetables at dinner (*p* = 0.01) and fruits (*p* = 0.04) at least once daily than women with lower educational attainment. Furthermore, a larger proportion of older women reported eating vegetables on sandwich at least daily (*p* = 0.01) than younger women (Table 2).

**Table 1:** Sociodemographic characteristics at inclusion among pregnant, nulliparous women (*N* = 575)

	n	%
Maternal age (years)		
< 20	7	1.2
20–24	136	23.7
25–29	263	45.7
30–34	128	22.3
35+	41	7.1
BMI (kg/m^2^)		
19.0- < 20.0^a^	60	10.4
20.0- < 25.0	347	60.3
25.0- < 30.0	125	21.8
30.0- < 35.0	30	5.2
≥ 35.0	13	2.3
Education (*N* = 574)		
< 7 years	0	0.0
7–10 years	9	1.6
10–12 years	74	12.9
Completed high school	97	16.9
< 4 years college/university	190	33.1
≥ 4 years college/university	204	35.5
Employment		
Employed	485	84.4
Student	50	8.7
Unemployed	22	3.8
Social welfare	11	1.9
Homemaker	7	1.2
Marital status		
Cohabiting with partner	553	96.2
Cohabiting with parents	7	1.2
Not cohabiting	15	2.6
Smoking		
Never	385	66.9
Previous	168	29.2
1–4 cig/day	13	2.3
5–9 cig/day	5	0.9
10–20 cig/day	4	0.7

^a^Women had to have BMI ≥19 kg/m^2^ to be included in the NFFD trial

**Table 2:** Changes in the frequency of fruit and vegetable consumption from pre-pregnancy to early pregnancy (*N* = 575)

	Pre-pregnancy		Early pregnancy^a^			
	%	95%CI	%	95%CI	∆ in %	*p*-value
Vegetables at dinner ≥ daily	22.2	17.9–26.6	22.3	17.9–26.7	0.1	0.75^†^
≤ 13 years education	17.1	10.8–23.5	17.4	11.0–23.8	0.1	
> 13 years education	27.4	21.8–32.9	27.2	21.6–32.8	0.2	0.81^‡^
BMI <25	20.7	15.9–25.4	21.5	16.8–26.3	0.8	
BMI ≥25	23.8	17.1–30.5	23.1	16.4–29.9	−0.7	0.39^‡^
Age < 25	21.1	14.0–28.3	20.8	13.6–28.0	−0.3	
Age ≥ 25	23.3	18.5–28.2	23.8	18.9–28.8	0.5	0.70^‡^
Vegetables on sandwich ≥ daily	13.2	9.5–16.9	17.2	13.2–21.2	4.0	<0.01^†^
≤ 13 years education	13.1	7.7–18.4	18.3	12.5–24.2	5.2	
> 13 years education	13.3	8.7–18.0	16.0	10.9–21.1	2.7	0.26^‡^
BMI <25	13.8	9.8–17.8	16.8	12.4–21.2	3.0	
BMI ≥25	12.7	7.0–18.3	17.5	11.3–23.7	4.8	0.40^‡^
Age < 25	9.6	3.5–15.6	11.7	5.0–18.3	2.1	
Age ≥ 25	16.8	12.7–21.0	22.7	18.1–27.2	5.9	0.12^‡^
Other vegetables ≥ daily	10.8	7.5–14.0	14.4	10.7–18.2	3.6	0.01^†^
≤ 13 years education	9.5	4.7–14.2	12.3	6.8–17.8	2.8	
> 13 years education	12.1	7.9–16.2	16.6	11.8–21.4	4.5	0.54^‡^
BMI <25	12.6	9.0–16.2	16.0	11.9–20.1	3.4	
BMI ≥25	8.9	3.8–14.0	12.9	7.1–18.7	4.0	0.83^‡^
Age < 25	11.5	6.0–16.9	13.8	7.6–20.0	2.3	
Age ≥ 25	10.1	6.4–13.8	15.1	10.8–19.3	5.0	0.38^‡^
Fruits^b^ ≥ daily	23.8	19.2–28.3	41.2	36.1–46.2	17.4	<0.01^†^
≤ 13 years education	20.4	13.8–27.0	36.2	28.9–43.6	15.8	
> 13 years education	27.1	21.4–32.9	46.1	39.7–52.5	19.0	0.46^‡^
BMI <25	25.9	20.9–30.8	46.1	40.6–51.6	20.2	
BMI ≥25	21.7	14.7–28.7	36.2	28.4–44.0	14.5	0.15^‡^
Age < 25	20.9	13.4–28.3	38.3	29.9–46.6	17.4	
Age ≥ 25	26.7	21.6–31.8	44.1	38.4–49.7	17.4	0.99^‡^
Other fruits^c^/berries ≥ daily	7.7	4.8–10.6	15.0	11.4–18.7	7.3	<0.01^†^
≤ 13 years education	8.9	4.8–13.0	16.2	10.8–21.6	7.3	
> 13 years education	6.5	2.9–10.2	13.8	9.2–18.5	7.3	0.99^‡^
BMI <25	9.6	6.5–12.7	16.6	12.6–20.6	7.0	
BMI ≥25	5.8	1.4–10.2	13.5	7.8–19.1	7.7	0.80^‡^
Age < 25	6.7	2.0–11.4	15.2	9.1–21.2	8.5	
Age ≥ 25	8.7	5.5–11.9	14.9	10.7–19.0	6.2	0.43^‡^
Fruits/vegetables as snacks ≥ daily	13.9	10.2–17.6	27.9	23.1–32.6	14.0	<0.01^†^
≤ 13 years education	12.8	7.4–18.1	25.6	18.7–32.6	12.8	
> 13 years education	15.1	10.4–19.8	30.1	24.1–36.2	15.0	0.59^‡^
BMI <25	15.8	11.8–19.8	31.1	25.9–36.3	15.3	
BMI ≥25	12.0	6.3–17.7	24.7	17.3–32.0	12.7	0.51^‡^
Age < 25	13.5	7.4–19.6	23.0	15.2–30.8	9.5	
Age ≥ 25	14.4	10.2–18.5	32.7	27.4–38.1	18.3	0.04^‡^

*BMI* Body Mass Index

^a^gestational week 15

^b^ apples, pears, oranges or bananas

^c^ other fruits than apples, pears, oranges or bananas

^†^ *P*-value based on repeated measure model

^‡^ *P*-value: Multilevel linear mixed model, including maternal education, BMI and age and the interaction terms: time*education, time*BMI and time*age

### Consumption of fruits and vegetables (FV)

Changes in FV consumption from pre-pregnancy to early pregnancy are presented in Table 2. The percentage of women eating vegetables on sandwiches (13 vs. 17%, *p* <0.01), other vegetables (11 vs. 14%, *p* = 0.01), fruits (24 vs. 41%, *p*<0.01), other fruits and berries (8 vs. 15%, *p* <0.01), and fruits and vegetables as snacks (14 vs. 28%, *p* <0.01) daily or more frequently increased significantly from pre-pregnancy to early pregnancy. The percentage of women who reported at least daily consumption of vegetables with dinner (22% at both time points) was stable (Table 2).

A larger proportion of older women reported a significant increase of at least daily consumption of “vegetables and fruits as snacks” from pre-pregnancy to gestational week 15 (14 vs. 33%) compared to younger women (14 vs. 23%) (interaction time^*^age, *p* = 0.04). There were no significant interactions between neither BMI nor maternal education and changes in intake of FV pre-pregnancy to gestational week 15 (Table 2).

## Discussion

The present study showed that the proportion of women consuming FV daily or more frequently increased from pre-pregnancy to early pregnancy independent of educational level and pre-pregnant BMI. However, there was a rather strong sociodemographic gradient in the consumption of FV, as more women with high educational level reported the highest consumption frequencies. From pre-pregnancy to early pregnancy, the proportion of women consuming FV daily or more frequently increased significantly in the categories: “vegetables on sandwich”, “other vegetables” and “fruit and vegetables as snacks”. From pre-pregnancy to early pregnancy the highest increment in consumption frequency of FV was reported in the categories “fruits” (apples, pears, oranges or bananas) and “fruits or vegetables as snacks”. Furthermore, the most frequent way of consuming vegetables was “vegetables at dinner”.

A low intake of FV may be explained by a variety of factors such as cost [19], availability, familiarity, and time for preparation [37–39]. Worldwide, higher socio-economic status tends to be associated with healthier food choices [40, 41]. In line with others, we found that a high intake of FV was associated with older age [8, 32–35, 42–44] and higher educational attainments [36, 42].

Maternal diet pre-pregnancy might influence implantation, placentation and embryogenesis [45, 46]. Thus, an increase in intake of various nutrients, including protein, folate, calcium, vitamin C and vitamin D, is recommended [47]. It is a concern that the present study seems to indicate a low consumption of FV pre-pregnancy. Similarly, Blumfield et al. found no evidence that women trying to conceive increased their consumption on nutrient rich foods such as FV [48]. In line with our results, several studies have found that only a small proportion of women consume the recommended number of vegetable servings per day, both pre-pregnancy [48] and during pregnancy [18, 35, 36, 44, 48, 49]. A national study from Australia showed that only 10% of pregnant women reported an intake of vegetables at or above recommendations [49]. Rodriguez-Bernal et al. reported that 47% of Spanish women had an intake of vegetables which was below recommendations in first trimester of pregnancy [43], while a Finnish study found that only between 16 and 30% of pregnant women consumed the daily recommendations of FV [44].

The observed increase in proportion of women who consume FV more frequently from pre-pregnancy to early pregnancy in the present study is in line with a recent study from Singapore which found that 46 to 67% of the women increased their consumption of FV from pre-pregnancy to late second trimester of pregnancy [24]. Further, the reported large and significant increase in the consumption frequency of fruits from pre-pregnancy to early pregnancy is in accordance with other studies [21, 23, 24]. Crozier et al. found that intake of fruits other than citrus fruit increased from pre-pregnancy to gestational week 11 [21]. However, they reported little change in the overall intake of FV from pre-pregnancy to early pregnancy, as the proportion of women consuming less than the recommended five portions of FV was 46% pre-pregnancy and 47% in late first trimester of pregnancy [21]. Fruits are often found to be more commonly consumed than vegetables, and daily recommendations are more frequently reached in pregnancy [18, 35, 36, 48, 49] compared to vegetables. Reported fruit intake is nonetheless varied: Malek et al. reported that 56% of the pregnant women adhered to recommendations regarding fruit intake [49], whereas in a cohort of younger Australian women 82% failed to meet recommendations [48]. One reason could be that vegetables often need more preparation and cooking than fruits. However, this discrepancy between vegetable and fruit consumption has been reported to be less pronounced or absent in areas with a traditional food culture containing more plant-based food [43].

Our finding that a higher proportion of older women increased their consumption of vegetables and fruits as snacks from pre-pregnancy to early pregnancy, compared to younger women is consistent with other studies reporting healthier food choices to be associated with increasing maternal age [8, 11, 32–35]. In the present study there were no associations between level of education and increase in the frequency of consumption of FV from pre-pregnancy to early pregnancy. Similarly we did not identify any association between BMI and increase in frequency of FV consumption. Women with higher educational level have in other studies been found to adhere to a healthier diet [18], and to consume more FV than women with lower educational level [17, 32, 35, 50, 51]. Furthermore, a healthier diet with a high content of fruit and vegetables are more commonly found among women with normal weight than those with overweight and obesity [18, 32]. Even though pregnancy is looked upon as a time were women are motivated towards positive lifestyle changes, an Australian qualitative study reported that a high proportion of pregnant women regarded pregnancy as a difficult period to change to a healthier lifestyle [52]. They point to pregnancy complications such as nausea, tiredness and cravings as possible barriers.

Most FV are high in fibre and have a low glycaemic index. Foods with lower glycaemic index produce fewer and smaller postprandial glucose episodes which may decrease subsequent hunger and total energy intake and prevent weight gain [53]. Further, FV are important sources of dietary compounds such as minerals, and a diversity of bioactive substances, including antioxidants and phytochemicals [54]. Dietary patterns comprising ample FVs are associated with decreased plasma concentrations of biochemical markers of endothelial dysfunction and inflammation [55–57]. Favourable health effects might operate through a positive modulation of the gut microbiota [58], promoting an increased bacterial richness, which has been inversely associated with insulin sensitivity and inflammatory markers [59]. Fertile women in Norway are reported to have an intake of fibre, vitamin D, folate and iron below recommended amounts [16]. Recommended daily intake of fibre is 25–35 grams per day, whereas the average intake of fibre among Norwegian women was 22 g per day in 2013 [16]. Around one in two fertile women are not aware the Norwegian Directorate of Health’s recommendations for a healthy diet [16]. Increasing intake of FV both pre-pregnancy and in pregnancy are important to improve fertile women’s diet. This study shows that pregnant women change to a healthier diet, although there is potential for further improvement. As pre-pregnancy diet is an important predictor for diet during pregnancy as well as for pregnancy outcome and future health for the mother and child, future interventions should focus on women planning, or at risk for, pregnancy.

### Strengths and limitations

The inclusion of women from public clinics attended by most of the pregnant women in Norway as part of the national antenatal care programme, and the high response rate are major strengths. Our data were to a large extent collected electronically, which is shown to be more valid than data collected by interviewer or paper questionnaire [60]. The present dataset has few missing data as the participants had to answer each question to progress in the questionnaire.

The FFQ used in the present study was primarily designed to capture changes in diet related to the intervention in the NFFD study and not to measure the specific or absolute intake of FV. The FFQ contained one item covering fruits with the highest intake in the Norwegian population (apples, oranges, pears or bananas) [17]. Intake of berries is low in most populations, berries are often not analysed as a separate entity, but are instead combined with fruits. In line with this, one FFQ item in the present study covered fruits or berries other than apples, oranges, pears or bananas. Further, three FFQ items covered solely vegetable consumption. However, since one FFQ-item asked about “fruits and vegetables as snacks”, we were not able to describe the exact frequency of the consumption of vegetables and fruits. The rather comprehensive FFQ used in the NFFD study requires a certain level of cognitive function to complete, and might not represent the best approach for including respondents with poor reading skills. As the respondents were included in an intervention study aiming at optimising nutrition and physical activity, they might have over-reported their intake of FV.

The study was limited to nulliparous women, and was biased towards older age and higher educational attainment. Furthermore, the women in the NFFD study were mainly white European [26]. In Norway, 48% of women between 25 and 29 years had not attended university in 2011 [61], compared to 28% of the women in the present study. Mean age of Norwegian nulliparous women at delivery was 28.2 years in 2011 [62], whereas mean age at inclusion in the present study was 28.1 years. This might reduce the external validity and the reproducibility of our results.

Although the present study was done in a relatively homogenous population, participants might define FV differently. In an American study, adults from a diverse ethnic background reported significant differences on classification of a number of food groups, including FV [63], and potatoes were often included in the vegetable category [63]. The FFQ-item “vegetables at dinner”, might not capture mixed vegetable dishes, and thus underestimate intake of vegetables. In line with the Norwegian Public Health dietary guidelines [15], potatoes were not included in the vegetable category in the present study. The FFQ-item on potatoes was placed before the FV items [25], minimizing the probability of reporting potatoes as vegetables. As the questionnaire was in Norwegian or English, few immigrant women attended the present study. Some immigrants are known to be at risk of low FV consumption [34], while other immigrant groups have a higher intake of vegetables than non-immigrants [34, 43].

Other limitations are the cross sectional design and the reliance on self-reported data. The data on pre-pregnancy diet was collected in retrospect, and thus we cannot rule out recall bias. The women who consented to participate in the NFFD trial might have been more health-conscious and more likely to adhere to a healthy lifestyle, including an increased intake of FV, than the average pregnant woman. We did not ask about motivation for changing dietary habits. The increase in FV consumption might have been due to public health recommendations or symptoms specific to pregnancy such as cravings, tiredness or nausea [64]. Since the women were recruited at their first visit to the antenatal clinic, nutritional advice from health personnel is less likely to have influenced the change. Seasonal variations have been described in intake of FV [65]. We did not take this into account. The present study was conducted in a country where a small proportion of the population is consuming the recommended amount of FV [17]. In populations with a higher intake the social gradients in consumption might have been less distinct [66, 67].

## Conclusion

From pre-pregnancy to early pregnancy we found an increase in the proportion of women consuming FV daily or more frequently. As pre-pregnancy diet is an important predictor for diet during pregnancy as well as for pregnancy outcome and future health for the mother and child, future interventions should focus on women planning, or at risk for, pregnancy.

## Abbreviations

BMI: Body Mass Index
CI: Confidence interval
FFQ: Food frequency questionnaire
FV: Fruits and vegetables
GA: Gestational age
MoBa: Norwegian Mother and Child Cohort Study
NFFD: Norwegian Fit for Delivery
SD: Standard deviation
